# Six Cases of Diarrhœa Treated with Monesia

**Published:** 1842-04

**Authors:** Q. Gibbon

**Affiliations:** Salem, N. J.


*Six Cases of Diarrhœa treated with Monesia.* By Q. Gibbon, Salem, N. J.—Case 1st.—Mrs. K.—Married. Had labored under chronic diarrhœa accompanied with ulceration of the intestines for two years. Three or four months ago she was put upon small doses of acet. of morphia, which, after several weeks continuance, arrested the disease, and the patient remained for two months apparently perfectly well. At the expiration of this time the disease suddenly returned, and soon regained all its former activity. The extract of monesiæ was now commenced in doses of 5 grs. three times a day, and afterwards increased to 7 grs. four times per day, and continued for ten days, but without any other benefit than a slight decrease of the discharge for the first two days. The medicine was then discontinued, and the morphia substituted with its former success.

Case 2d.—A child of W. H., affected with diarrhœa of 18

months standing, had used anodynes, alteratives and astringents freely, but without permanent benefit. The ext. monesiæ given in doses of two grs., three times per day, assisted by the warm bath and frictions with flannel, checked the discharges in two days, and removed all traces of the disease in two weeks.

Case 3d.—J. M., aged 70—health delicate, had a diarrhœa, with short intermissions, for six months—had been treated with creta cum hydrarg. with but temporary relief. Took six grs. of monesia four times per day, from the 2d to the 10th of November, assisted by a few grains of pulv. doveri., occasionally, at bed time. The discharge ceased in two days, and upon the tenth he expressed himself perfectly well.

Case 4th.—Mrs. J.—Diarrhœa of four or five days continuance—slight fever, tormina, stools bloody and slimy. Had resorted to no previous treatment. Took 8 grs. of ext. mones. three times the first day, which checked the discharge, and allayed all unpleasant symptoms.

Case 5th.—A child of four years of age, affected with a bowel complaint which had commenced in the summer, and continued unchecked until November, though treated with a variety of astringents, and subjected to counter-irritation upon the abdomen. The monesia was given in doses of 2 grs. five times daily for six days, with the effect of gradually diminishing the discharges, changing their color and consistence, and creating a vigorous appetite, which the child had not previously enjoyed. Having no more of the remedy I was compelled to relinquish it. Dover's powder and flannel frictions were substituted, and I had the satisfaction of seeing my patient perfectly relieved in another week.

Case 6th.—In this case the remedy entirely failed as in the first case. It was one of diarrhœa supervening upon phthisis pulmonalis, and had been previously treated by creta cum opio., acet. of lead, acet. of morphia, and several of the vegetable astringents without benefit. The monesia was prescribed at first to the amount of 15 grs. and afterwards increased to 40 grs. per day, and persevered in for three weeks—when, as no abatement of the symptoms was perceived, the remedy was discontinued at the request of the patient.

Remarks.—That the above cases may be entitled to their just weight in the evidence now accumulating both for and against this new remedy, it may be well to state, that the few last doses taken by Mrs. K. in the first case, occasioned slight nausea accompanied by a slight burning in the stomach. With this exception, I have not witnessed any of the irritating effects detailed by St. Ange. My doses, however, were not so large as he prescribed. The above cases lead me to infer that monesia is more astringent than tonic in its operation.—*Am. Med. Intelligencer.*

